# Radiation recall after capecitabine in a patient with recurrent nasopharyngeal carcinoma: a case report

**DOI:** 10.1186/s13256-016-1033-1

**Published:** 2016-09-07

**Authors:** Victor Lee, Ka-On Lam, Dora Kwong, To-Wai Leung

**Affiliations:** 1 Department of Clinical Oncology, Queen Mary Hospital, Li Ka Shing Faculty of Medicine, The University of Hong Kong, Hong Kong, Hong Kong; 2 1/F, Professorial Block, Queen Mary Hospital, 102 Pokfulam Road, Hong Kong, Hong Kong

**Keywords:** Capecitabine, Intensity-modulated radiation therapy, Nasopharyngeal carcinoma, Radiation recall

## Abstract

**Background:**

Capecitabine has been commonly used in recurrent or metastatic nasopharyngeal carcinoma. However, radiation recall after capecitabine for nasopharyngeal carcinoma has not been reported.

**Case presentation:**

We report the case of a 64-year-old Chinese woman with locoregionally advanced nasopharyngeal carcinoma previously treated with induction chemotherapy followed by concurrent chemoradiation 6 years ago. She developed cervical, mediastinal, and abdominal nodal relapses 14 months later. She then received capecitabine with initial excellent tumor response for 1 year but disease recurrence was noticed at the peripancreatic nodal region, which was successfully treated with concurrent chemoradiation with capecitabine. Unfortunately, she developed progressive erythema of the face and neck region at exactly the previous irradiation site for her initial nasopharyngeal carcinoma, 2 months after taking capecitabine. She initially ignored it, but it became more confluent and serious. Eventually, a facial skin biopsy was performed showing nonspecific chronic inflammation only. The diagnosis was most likely radiation recall phenomenon since capecitabine was the only drug she received before development of this dermatological manifestation on her previously irradiated face and neck. Treatment was conservative and supportive albeit with no significant clinical improvement.

**Conclusions:**

Radiation oncologists should be aware of this potential risk of capecitabine, especially when it is administered for a long period of time.

## Background

Radiation recall is a rare and acute dermatological condition typically occurring at the site of previous radiation therapy, after administration of a certain type of oral or intravenous medication particularly chemotherapeutic agent(s) or, less likely, targeted therapies [1–4]. Skin is the most common site of manifestation with incidence between 6 and 11 % according to previous literature [5]. The time lapse between previous radiation therapy and the onset of radiation recall after use of the causative drug varies, ranging from days to months or even many years after the last radiation therapy [2, 4–6]. It has also been recently postulated that the severity of radiation recall is associated with the dose of the medication. Treatment would be most likely conservative with modest improvement after use of topical or systemic glucocorticoids, but the condition may persist even if the causative drug is withdrawn, especially when there is irreversible damage to the dermis.

Capecitabine, a prodrug of 5-fluorouracil, has been commonly employed in the treatment of breast cancer, upper and lower gastrointestinal cancer, and pancreatic cancers and thus all patients who were previously reported to suffer from radiation recall after capecitabine had their primary tumors in these tracts [7–11]. Capecitabine, in fact, is also active for nasopharyngeal carcinoma (NPC), an endemic disease in Southern China including Hong Kong, as shown in previous phase II and III studies [12–16]. Some case reports were published on radiation recall in patients previously treated for their head and neck cancers [17–19]. To the best of our knowledge, no report has been published on radiation recall after use of capecitabine for NPC. We here report the case of a patient who suffered from radiation recall phenomenon at her faciocervical region previously irradiated for her NPC, after taking capecitabine for her more recently developed cervical, mediastinal, and abdominal nodal relapse 1 year after radical chemoradiation for her NPC.

## Case presentation

**Fig. 1:**
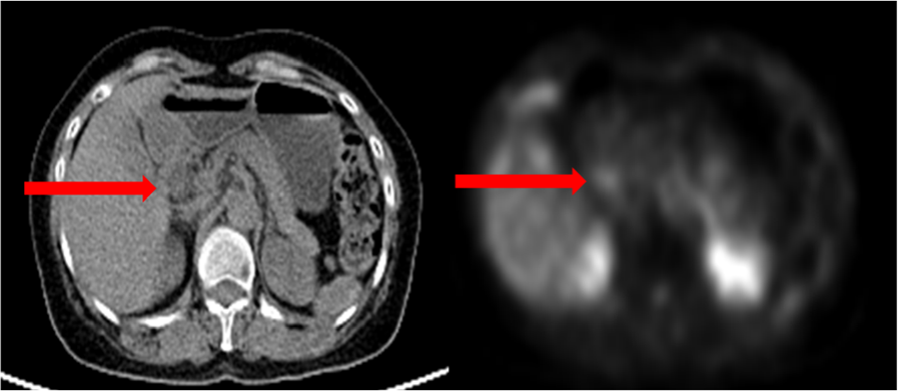
Positron emission tomography with integrated computed tomography image showing the hypermetabolic peripancreatic nodal relapse (*red arrow*)

**Fig. 2:**
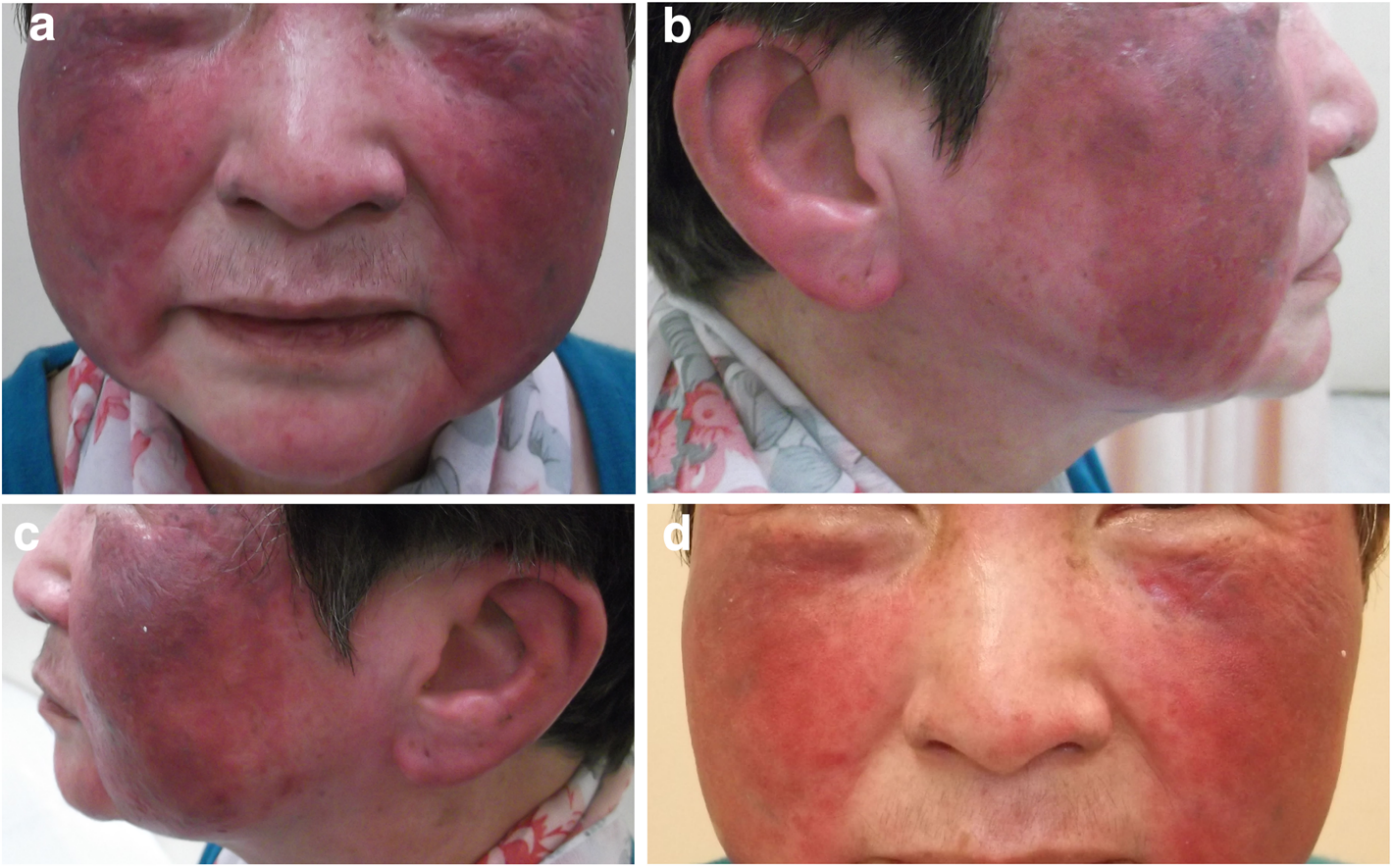
Radiation recall phenomenon after capecitabine for our patient who suffered from recurrent nasopharyngeal carcinoma. **a** Forehead and face. **b** Right face and ear. **c** Left face and ear. **d** Persistent appearance after laser therapy

A 64-year-old Chinese woman was diagnosed to have stage IVB T1N3bM0 undifferentiated type of nasopharyngeal carcinoma (NPC) 6 years ago. Pretreatment plasma Epstein-Barr virus (EBV) deoxyribonucleic acid (DNA) was 827 copies/mL. She received induction chemotherapy consisting of one cycle of cisplatin and 5-fluorouracil and two more cycles of carboplatin (area-under-the-curve value 5) plus 5-fluorouracil in view of her deteriorating renal function, followed by radical chemoradiation using carboplatin concurrent with intensity-modulated radiation therapy (IMRT) with 70 Gy in 33 fractions to the gross primary tumor and neck nodes and 66 Gy in 33 fractions to the high-risk primary and neck nodal regions, all over 6.5 weeks delivered by Simultaneous Accelerated Radiation Therapy (SMART) technique completed 5 months later. Posttreatment plasma Epstein-Barr virus deoxyribonucleic acid (EBV DNA) became undetectable at 8 weeks after IMRT. Unfortunately, her disease relapsed 14 months later with lymph node metastases to the left supraclavicular fossa and the right superior mediastinal region, together with a distant lymph node metastasis at the peripancreatic region revealed by [18 F]fluorodeoxyglucose positron emission tomography with integrated computed tomography (PET-CT) (Fig. 1). Plasma EBV DNA rose to 338 copies/mL. In view of distant metastasis, she started capecitabine at 1000 mg/m^2^ twice daily from day 1 to 14 and every 3 weeks afterward and titrated up to 1250 mg/m^2^ twice daily from day to 1 to 14 every 3 weeks from cycle 3 onward with a significant drop of plasma EBV DNA to 0 copies/mL after taking capecitabine for 7 months. She continued capecitabine up to 1 year until further disease progression with increasing in size of the peripancreatic lymph node metastasis with plasma EBV DNA elevation to 64 copies/mL. In view of the solitary peripancreatic nodal disease, she received conformal radiotherapy (50 Gy in 25 fractions over 5 weeks) to the peripancreatic lymph node concurrent with capecitabine 825 mg/m^2^ twice daily and 5 days a week on radiotherapy days only. Plasma EBV DNA dropped to 0 copies/mL and her PET-CT scan 9 months later confirmed complete metabolic response of the peripancreatic lymph node. Capecitabine was then stopped after the complete response demonstrated in this latest PET-CT scan. Unfortunately, she noticed that her face gradually developed progressive erythema and increasing warmth with relative sparing of the nostrils and perinasal region 2 months after taking capecitabine. It was initially ignored by our patient until the skin erythema became more confluent and prominent at the irradiation site of the previous IMRT for her initial NPC (Fig. 2a-c). It did not subside with capecitabine cessation as well as application of emollients, topical and oral steroids, and even antibiotics. A skin biopsy showed nonspecific inflammation only. As the dermatological manifestation was confined to her face and neck only and she had not received any other oral or topical medication except capecitabine, radiation recall was the most likely underlying diagnosis to account for her current skin condition. She received a course of laser therapy prescribed by a private dermatologist but the skin condition prevailed (Fig. 2d). Unfortunately, her facial skin condition has been persistent for more than 4 years though no further evidence of NPC recurrence has been noted so far.

## Discussion

Capecitabine is rarely used as part of systemic chemotherapy in head and neck squamous cell carcinoma but it is more commonly used in breast, esophageal, gastric, colorectal, and pancreatic cancers [7–11]. On the other hand, capecitabine is one of the most commonly prescribed regimes for recurrent and metastatic NPC, a separate disease entity from head and neck squamous cell carcinoma based on its different etiology and geographical epidemiology, since the response rate ranges from 23.5 % as monotherapy to 62.5 % when used in combination with cisplatin [12–15]. More recently, capecitabine has also been found efficacious as induction chemotherapy followed by concurrent chemoradiation in a phase III randomized controlled trial conducted in Hong Kong [16]. Radiation recall related to prior use of capecitabine has been reported in patients with breast and pancreatic cancers [20–22]. However, it has not been reported so far in head and neck cancers, including NPC. The exact pathophysiology for radiation recall phenomenon remains to be deciphered. Vascular damage, epithelial stem cell sensitivity or hypersensitivity to chemotherapeutic agents have been postulated as the underlying mechanisms [5, 23]. More recently, thymidine phosphorylase, a potent angiogenic factor was shown to be associated with the development of radiation recall after capecitabine use [22, 24]. Sawada *et al.* revealed that external radiotherapy induces thymidine phosphorylase and it enhanced the efficacy of capecitabine in human cancer xenografts [25]. Thymidine phosphorylase produces 2-deoxy-D-ribose-1-phosphate during thymidine catabolism, which in turn generates oxygen radical species during the early stages of protein glycation. It was suggested that thymidine led to oxidative stress in thymidine phosphorylase-overexpressing cancer cells, leading to production of stress-induced angiogenic factors, vascular endothelial growth factor, and interleukin-8 and induced matrix metalloproteinase 1, accounting for thymidine phosphorylase-induced angiogenesis [22, 25]. Upregulation of thymidine phosphorylase by previous external radiotherapy gave rise to development of angiogenesis in the previously irradiated region, leading to hypervascularity and erythema. In fact, the well-recognized palmar-plantar erythrodysethesia (hand-foot syndrome) as a common side effect of capecitabine, may be due to this hypervascularity sequela. Since our patient had received capecitabine continuously for more than 1 year, the accumulative dose of capecitabine might predispose to the development and persistence of her radiation recall. Treatment for radiation recall is most largely conservative with close surveillance. The subsequent irreversible damage of the underlying dermis brought about by prolonged exposure to capecitabine may explain her poor response to symptomatic treatment. Medication like moisturizers, antihistamines or emollients may alleviate symptoms of desquamation, itchiness or warmth. Topical or systemic glucocorticoids may help reduce the inflammatory response but they are usually not curative. Laser therapy, as our patient received as per self-intention, was not proven effective in alleviating the skin condition. Response rates to these symptomatic treatment have been poorly understood as they heavily depend on the pharmacokinetics of the causative agent, duration of use of the causative agent, and whether irreversible skin damage has occurred or not [2, 5]. Usually the recall reaction may resolve more rapidly after discontinuation of intravenous agents rather than oral treatment. Some reactions to intravenous agents may improve within hours. However, it may takes weeks, months or even longer for the recall reaction to resolve, especially if the causative agent is an oral medication [2].

## Conclusions

In summary, this is the first case of radiation recall related to capecitabine in a patient with recurrent NPC. Radiation oncologists should be vigilant of this potential complication associated with capecitabine especially when it is used for a long period of time. Treatment is mainly supportive though significant clinical improvement is not expected.

## Abbreviations

DNA: Deoxyribonucleic acid
EBV: Epstein-Barr virus
IMRT: Intensity-modulated radiation therapy
NPC: Nasopharyngeal carcinoma
PET-CT: Positron emission tomography with integrated computed tomography
SMART: Simultaneous Accelerated Radiation Therapy
